# Challenges in the clinical development pathway for triple and multiple drug combinations in the treatment of uncomplicated falciparum malaria

**DOI:** 10.1186/s12936-022-04079-9

**Published:** 2022-02-22

**Authors:** Quique Bassat, Oumou Maïga-Ascofaré, Jürgen May, Jerôme Clain, Ghyslain Mombo-Ngoma, Mirjam Groger, Ayôla A. Adegnika, Jean-Claude Dejon Agobé, Abdoulaye Djimde, Johannes Mischlinger, Michael Ramharter, ASAAP and Multimal Consortia

**Affiliations:** 1 ISGlobal, Hospital Clínic - Universitat de Barcelona, Barcelona, Spain; 2 Centro de Investigação em Saúde de Manhiça (CISM), Maputo, Mozambique; 3 ICREA, Passeig de Lluís Companys 23, 08010 Barcelona, Spain; 4 Pediatrics Department, Hospital Sant Joan de Déu, Universitat de Barcelona, Esplugues, Barcelona, Spain; 5 Consorcio de Investigación Biomédica en Red de Epidemiología y Salud Pública (CIBERESP), Madrid, Spain; 6 Kumasi Center for Collaborative Research, Kumasi, Ghana; 7 Bernhard Nocht Institute for Tropical Medicine, Hamburg, Germany; 8 German Center for Infection Research, Partner Site Hamburg- Lübeck-Borstel-Riems, Hamburg- Lübeck-Borstel-Riems, Germany; 9 Université de Paris, Centre National de Référence du Paludisme, Hôpital Bichat- Claude Bernard, Assistance Publique des Hôpitaux de Paris, Paris, France; 10 Centre de Recherches de Lambaréné, Lambaréné, Gabon; 11 Institute for Tropical Medicine, University of Tübingen, Tübingen, Germany; 12 Malaria Research and Training Centre (MRTC), Faculty of Pharmacy, Université des Sciences, des Techniques et des Technologies de Bamako (USTTB), Bamako, Mali

**Keywords:** Malaria, Artemisinin-based combination therapy, Falciparum, Regulatory pathway

## Abstract

The addition of a third anti-malarial drug matching the pharmacokinetic characteristics of the slowly eliminated partner drug in artemisinin-based combination therapy (ACT) has been proposed as new therapeutic paradigm for the treatment of uncomplicated falciparum malaria. These triple artemisinin-based combination therapy (TACT) should in theory more effectively prevent the development and spread of multidrug resistance than current ACT. Several clinical trials evaluating TACT—or other multidrug anti-malarial combination therapy (MDACT)—have been reported and more are underway. From a regulatory perspective, these clinical development programmes face a strategic dilemma: pivotal clinical trials evaluating TACT are designed to test for non-inferiority of efficacy compared to standard ACT as primary endpoint. While meeting the endpoint of non-inferior efficacy, TACT are consistently associated with a slightly higher frequency of adverse drug reactions than currently used ACT. Moreover, the prevention of the selection of specific drug resistance—one of the main reasons for TACT development—is beyond the scope of even large-scale clinical trials. This raises important questions: if equal efficacy is combined with poorer tolerability, how can then the actual benefit of these drug combinations be demonstrated? How should clinical development plans be conceived to provide objective evidence for or against an improved management of patients and effective prevention of anti-malarial drug resistance by TACT? What are the objective criteria to ultimately convince regulators to approve these new products? In this Opinion paper, the authors discuss the challenges for the clinical development of triple and multidrug anti-malarial combination therapies and the hard choices that need to be taken in the further clinical evaluation and future implementation of this new treatment paradigm.

## Background

Since the Second World War monotherapy constituted the universal treatment paradigm for falciparum malaria [1]. When drug resistance emerged in a geographical region (most frequently initially in South-East Asia), a new anti-malarial drug was evaluated in clinical trials against a failing first-line therapy demonstrating superior efficacy and ultimately replacing it as standard treatment for uncomplicated malaria [1–3]. This process occurred in similar ways for the major anti-malarials of the twentieth century—chloroquine, sulfadoxine-pyrimethamine, and mefloquine—until finally no replacement drug was readily available for clinical testing [4, 5]. In parallel, the spread of these drug resistant *Plasmodium falciparum* isolates throughout sub-Saharan Africa was paralleled by a dramatic increase in malaria-related mortality, highlighting the impact of drug resistance from a public health perspective [6].

To address this vicious cycle of drug development and emergence of drug resistance, and mimicking what had been historically proposed for other infectious diseases, such as tuberculosis (TB) or HIV, a new treatment paradigm emerged at the turn of the century by proposing artemisinin-based combination therapy (ACT) as standard of care for uncomplicated malaria [4]. ACT demonstrated higher cure rates than the hitherto failing first-line drug mefloquine. Over the following decade (and until today in most of the malaria endemic world outside of the Greater Mekong Region) ACT showed unprecedentedly high cure rates, favourable tolerability and safety and contributed importantly to the subsequent reduction of the global malaria burden.

## **Rationale for evaluation of triple artemisinin-based combinations therapy (TACT) and multidrug anti-malarial combination therapy (MDACT)**

Whereas it was initially hoped that ACT will prevent the further emergence of drug resistance due to the combination of two independently acting drugs, this assumption was unfortunately not substantiated [7]. Over the past two decades the emergence of *P. falciparum* strains insufficiently responsive to conventional ACT in the Greater Mekong Region has led to a challenging public health situation for this region [8–10]. The use of ACT is associated with unacceptably low cure rates in some regions and the registration of drugs belonging to novel classes of anti-malarials will still require several years [9–11]. To overcome this situation triple artemisinin-based combination therapy (TACT) has been proposed as new treatment paradigm to bridge this period of time until new drugs become licensed ensuring high cure rates for multidrug resistant falciparum malaria [11, 12].

The therapeutic concept of TACT is—at least in part—motivated by two factors. Firstly, insights into the molecular mechanism of anti-malarial drug resistance indicates counter-selection of drug resistance by piperaquine and mefloquine—two of the most widely used partner drugs. Secondly—and more importantly for the concept of other TACT and MDACT—the rationale of combining three (or more) anti-malarial drugs relies in more general terms on the conceptual framework of mutual protection of the long acting partner drugs sharing similar pharmacokinetic characteristics from the selection of multiple random drug resistance mutations [12] (Table 1).

**Table 1:** Rationale for the clinical development of Triple Artemisinin-based Combination Therapies

Rationale for TACT	Detailed information	Perspective for TACT clinical development plan
Clinical failure of ACT in the Greater Mekong Region	Artemisinin resistance—or delayed treatment response to artemisinins—is a concern in the Greater Mekong Region. If drug resistance against the partner drug emerges at the same time, ACT treatment is associated with unacceptably high rates of treatment failure. Adding a second partner drug to a failing ACT restores cure rates to high levels. This is facilitated by independent modes of action and in the case of mefloquine and piperaquine thought to be mediated by counter-selection of drug resistance markers.	Adding a single drug to a failing drug combination is thought to accelerate the selection of drug resistant mutants. TACT should therefore ideally be evaluated and implemented before drug resistance against ACT emerge on a large scale in an endemic region.
Prevention of emergence and spread of drug resistance by Triple Artemisinin Combination Therapies	The short but rapidly acting artemisinin derivative quickly reduces the total number of parasites circulating in a patient and thus reducing the stochiastic chances for selection of resistant mutants in the remaining low number of parasites.	This benefit of artemisinins is reduced in regions of delayed parasite clearance by artemisinins thus increasing the risk for selection of drug resistance. TACT should therefore ideally be implemented before the rapid activity of artemisinins is compromised.
	Adding a pharmacokinetically matched long acting partner drug to ACT leads to mutual protection of partner drugs against selection of drug resistance over the prolonged elimination period.	
	Combining three drugs with different modes of action reduces the likelihood for the selection of drug resistant mutants	
Priority patient population for TACT	According to the global epidemiology of falciparum malaria and its impact on mortality, African children are undoubtedly the most important patient population for new antimalarial treatments. Historically, it was also demonstrated in sub-Saharan Africa that drug resistance against previous first line antimalarials translated to a substantial increase in excess mortality.	Efficacy, tolerability, safety of TACT need to be evaluated in African children early on in the development plan.
	Efficacy, tolerability, and safety are not necessarily similar in adults as in children as well as in semi-immune and non-immune patients.	

The concept of TACT has been born out of necessity by failing artemisinin-based combinations in the Greater Mekong Region, while new drugs are not yet available on the market. However, it is understood that the full potential of TACT to prevent drug resistance is its use in settings where drug resistance against any of the partner drugs has not yet emerged as adding a drug to a failing regimen may accelerate the development of parasites resistant to all drugs combined in the anti-malarial combination [12]. Today the Greater Mekong Region harbours only low absolute numbers of falciparum infections, whereas sub-Saharan Africa is home to 94% of the global malaria burden [13]. If TACT and MDACT are to live up to their full potential, it is therefore understood that this new therapeutic concept needs to be introduced in sub-Saharan Africa before artemisinin-based combinations start to fail there. Given recent reports about *kelch-13* mutants associated with delayed parasite clearance times, the window of opportunity may be rather short on the African continent [14].

## Considerations on the efficacy outcomes of triple and multiple drug combinations for the treatment of malaria

In contrast to the historic situation where a new anti-malarial monotherapy was tested in a clinical trial aiming to demonstrate superior efficacy compared to a failing first-line drug, this situation changed with the development of the first artemisinin-based combinations. Subsequent combinations were then largely evaluated in clinical phase III trials aiming for the proof of non-inferiority of PCR corrected efficacy compared to the standard ACT. The clinical development of TACT now leads again to a paradigm shift as TACT constitute a new treatment paradigm that is developed with the intention to have high cure rates in settings of multidrug resistance, arguing for the requirement for superiority trials compared to standard ACT. However, to allow for TACT’s full potential in the prevention of drug resistance, the clinical development of TACT would ideally take place in settings where ACT remains efficacious. This is then a challenging situation from the perspective of a clinical development plan as TACT can so only be tested for non-inferiority of efficacy compared to standard ACT as primary endpoint. Non-inferiority of PCR corrected adequate clinical and parasitological response becomes the main efficacy outcome due to the continued high cure rates > 95% of the comparator ACT in the absence of drug resistance against artemisinins and their partner drugs [11, 15].

Conclusive evidence from clinical trials evaluating TACT in regions where ACT is currently failing is also not a feasible option due to the low number of malaria cases in the Greater Mekong Region. This low number precludes the recruitment of a sufficient number of patients to demonstrate superiority in efficacy in large clinical phase III trials. This fact was demonstrated by the first large clinical trial assessing TACT, which ultimately chose to enlarge the initial clinical trial network to include recruitment at sites outside of the Greater Mekong Region and thus switching from a superiority to a non-inferiority endpoint for efficacy analysis for most of the recruitment centres [11]. Whereas this change in recruitment strategy may be seen as a weakness for the primary efficacy analysis, it allowed at the same time to evaluate TACT in the most important patient population of interest – African children.

In summary, even if TACT is evaluated in regions of high prevalence of multidrug resistance, the low incidence of malaria in these regions precludes recruitment of sufficient numbers of patients for large clinical phase III trials. At the same time the theoretical rationale for TACT favours to primarily evaluate these therapeutic regimens in regions with high susceptibility to ACT and in African children. New TACT, therefore, needs to be evaluated in paediatric patients in sub-Saharan Africa to allow their safe use in this most important patient population. This necessary choice comes with the consequence of having to accept non-inferiority of efficacy as the primary pharmacodynamics endpoint.

## Considerations on the tolerability and safety outcomes of triple and multiple drug combinations for the treatment of malaria

Combining two or more drugs in a therapeutic regimen is inevitably leading to a higher number of adverse drug reactions compared to monotherapy as each compound comes with its own off-target effects. Clinical development programmes for TACT, therefore, need to be carefully designed to assess the frequency and severity of adverse events to allow for an informed appreciation of the side effect profile of a new TACT regimen besides pharmacokinetic drug-drug interactions. Most common tolerability findings may include rather unspecific findings of asthenia, or gastrointestinal disturbances including abdominal pain, nausea and vomiting or allergic drug reactions [11, 15]. As an unbiased quantitative estimation of tolerability is of high importance for the risk-benefit analysis of these therapeutic regimens compared to standard ACT, blinding of study patients and investigators to the treatment allocation is of high importance, even more so than in conventional drug development programmes.

At the same time a diligent safety assessment is of high importance as the combination of more than one drug may lead to anticipated and indeed unanticipated safety signals. The most common safety concerns involve drug induced hepatic injury and cardiac toxicity but other safety signals including renal, pancreatic, haematological findings as well as neurological side effects are of importance, too. It is recommended that these potential safety signals are investigated proactively and diligently by clinical assessments and diagnostic procedures as safety assurance arguably is of even higher importance in combination regimens than in monotherapy. Frequent safety signals may be detected in clinical phase I, II, and III trials. Rare, but potentially life-threatening safety events, may however only be reliably detected and their frequency estimated in large phase IV trials or post-marketing surveillance. This is a challenge that requires adequate attention to ensure safety assurance of new TACT in sub-Sahara Africa. In summary, currently reported clinical trials indicate that TACT will most likely result in slightly worse tolerability, while still demonstrating good safety profile [11]. Safety needs to be addressed proactively in clinical trials and in post-marketing registration studies to ensure maximum reassurance for the safe use of TACT.

## Ancillary endpoints in TACT clinical development programmes

Clinical development plans of TACT should include key outcomes supporting the rationale for the use of TACT. This includes the assessment of molecular markers for drug resistance at recruitment of patients into clinical trials as well as reassessment in case of re-appearance of parasitaemia or re-treatment. This analysis may allow for a better understanding of potential selection of drug resistant mutants. TACT may also have a substantial impact on the onward transmission of malaria by targeting the sexual developmental stages of *Plasmodium*, blocking the sexual cycle in the mosquito or by a direct lethal effects on the vector [16–18].

Clinical development plans should, therefore, include these important aspects to quantify any of these features of anti-malarial treatment. The prevention of the development of drug resistance and its spread by the vector is one of the key rationales for TACT, which is why direct evidence from these clinical trials becomes critical.

At the same time overenthusiasm in these ancillary analyses needs to be cautioned. It is highly unlikely that molecular analysis of drug resistance markers from a single clinical trial will lead to conclusive evidence for the prevention of drug resistance by TACT. This is due to the fact that only a small number of patients (usually < 10% of recruited participants) will experience re-appearing parasitaemia in high transmission regions due to the highly efficacious therapeutic regimens [11]. Contrary to other viral and bacterial infectious diseases, such as HIV and TB, selection of *de novo* drug resistance is overall a rare event in the eukaryote *P. falciparum*, probably also due to the rapid elimination of malaria parasites as compared to mycobacteria or HIV. Contrary to early HIV treatment trials indicating selection of drug resistance in virtually all individual patients, selection of anti-malarial drug resistance constitutes rare events potentially occurring only a few times within a decade in an entire population living under massive anti-malarial drug pressure in an endemic region [19, 20]. This is, therefore, not an event that can be reliably assessed in vivo in individual clinical trials or clinical development programmes. However, once a drug resistant mutant has emerged, it may spread much more quickly in malaria compared to HIV or TB due to malaria’s higher reproductive number [21]. Reporting of resistance markers is, therefore, important for future metanalytic approaches and mathematic modelling to assess the true preventive effect of triple and multiple drug combinations against the development and spread of drug resistant *P. falciparum* parasites.

In summary, the evaluation of ancillary endpoints such as the selection of molecular resistance markers and transmission blocking potential are of high importance and shall be incorporated in trials evaluating triple artemisinin-based combinations. At the same time, it is unlikely that single clinical trials and clinical trial programmes will lead to conclusive evidence in these key aspects of TACT use. The ultimate aspiration of TACT to prevent the development of drug resistance may only be appreciated in ecological studies, with in vivo and molecular drug resistance data available once this treatment concept has been implemented in large geographical settings for a sufficient period of time, or by pooling and meta-analysing the information generated through several different trials.

## TACT: a difficult perspective for funders, regulators, and manufacturers

As elaborated above, in the current situation triple artemisinin-based combinations are most likely to demonstrate non-inferior efficacy, slightly worse tolerability, and inconclusive evidence for their potential to prevent the development and spread of drug resistance while indicating the absence of frequent safety issues in currently ongoing clinical development programs. This constitutes a seemingly unattractive combination of key characteristics for a therapeutic product and thus potentially putting in question their further clinical development. Given that this also comes with inevitably increased costs of triple- and multidrug combinations, and the need for the development of new fixed dose combination formulations compared to conventional artemisinin-based combinations by adding another drug to the existing combination, this situation is indeed not the default case for funders, pharmaceutical companies, and regulators to happily embark on this new treatment concept. However, based on our understanding of the emergence and selection of anti-malarial drug resistance over the past century, TACT or in more general combination of three or more anti-malarials may exactly be what needs to be implemented to reduce the likelihood of rapid selection of drug resistance (Table 2).

**Table 2:** Challenges for the clinical development plans of Triple Artemisinin Combination Therapies and their impact

Challenges	Detailed information	Perspectives on clinical development plans
Efficacy evaluation	Clinical phase III trials need to be conducted in regions without high failure rate of ACT and need to include African children. Recruitment at sites in the Greater Mekong Region is encouraged but is limited due to low incidence of malaria in this region.	Non-inferiority of efficacy compared to standard ACT is most appropriate primary efficacy outcome. Superiority may be demonstrated in secondary sub-analysis of sites located in regions with high prevalence of multidrug resistance.
Tolerability evaluation	Treatment with more than one drug at therapeutic dosage inevitably leads to higher frequency and severity of off-target effects. TACT are therefore associated with (slightly) worse tolerability mostly concerning gastrointestinal side effects, nausea, vomiting.	Objective quantification of adverse events is of high importance for an informed comparative assessment of TACT compared to ACT. Study designs including blinding of patients and assessors are therefore encouraged.
Safety evaluation	Evaluation for potential safety signals is of high importance when combining multiple drugs. For antimalarials a special focus should be laid on evaluation of cardiotoxicity, hepatic and renal toxicity and haematological and neurological side effects. Frequent safety issues may be identified in phase I-III trials, whereas rare but potentially clinically important safety events may only be identified in phase IV studies.	Focused investigation of ECG changes, haematological and biochemical parameters should be proactively included in study protocols to provide reassurance that potential safety signals may be detected.
Drug interactions	Pharmacokinetic drug interaction is a key issue for multiple drug combinations. Drug-drug interactions may be evaluated in silico prior to clinical trials based on the known metabolic pathways. Focussed pharmacokinetic assessment in patients is however also of high importance to allow for evaluation of increased or decreased drug exposure in vivo. Potential for drug interaction also includes the modification of efficacy, tolerability and safety by the interaction of several drugs. These pharmacodynamic drug interactions need to be assessed as detailed above.	Focused assessment of pharmacokinetic characteristics of TACT in rich and/or population pharmacokinetic assessment is of high importance and should be added early on in the clinical development plan.
Prevention of drug resistance	The effective prevention of the emergence and spread of drug resistance is one of the main rationales for TACT. Molecular (and phenotypic) drug resistance monitoring of parasites at recruitment and at time of reappearance is therefore of importance to assess whether TACT select for drug resistance or prevent it.	Realistically, these molecular investigations will not lead to clear and definitive outcomes due to the rare event of selection of drug resistance by antimalarials. However, inclusion of these components in study protocols is of importance to build up a body of evidence which may lead to conclusive evidence in the long run by meta-analysis of multiple trials and mathematic modelling. For this purpose public sharing of these data will be of high importance.
Transmission blocking potential	Reducing onwards transmission of malaria by antimalarials drugs may be an effective way of preventing the spread of drug resistance. In case of preclinical evidence for transmission blocking potential for one of the drugs, focused assessment on transmissibility is highly informative.	Focused assessment on impact on transmission shall be included in clinical trial protocols whenever justified and feasible. Again it is unclear whether individual clinical trials will provide conclusive answers but pooling of data from several trials may provide important information on this aspect of antimalarial chemotherapy.
Drug formulation of TACTs	Fixed dose combinations have several advantages compared to loose combinations including better adherence to the recommended regimen. This necessitates the administration of each drug at the same time points as the overall TACT which may be challenging when combining drugs that are administered twice daily with those administered only once daily. Also child friendly drug formulations have importantly improved the management of young children and should therefore be a priority in the clinical development of TACTs. Currently no fixed-dose or child-friendly drug formulation of TACTs is under clinical investigation.	Once TACTs are shown to be safe, well tolerated and efficacious, fixed dose drug combinations will be required for roll-out in clinical routine. For this purpose bioequivalence studies will be needed to bridge data from large clinical phase III trials with pharmacokinetic data from fixed-dose formulations.
Cost effectiveness of TACT	Addition of a third drug to a two-drug combination inevitably increases cost of the treatment. It is challenging to factor in cost savings by delaying the development of drug resistance.	Information about cost of goods and cost for individual TACTs will constitute important information for public health decision makers.
Clinical phase IV studies and post-marketing surveillance	Rare but clinically important safety signals may only be detected in large phase IV trials and post-marketing surveillance.	Provision for systematic data collection and reporting as soon as TACT will be deployed on a large scale.
Perspective for funders, pharmaceutical companies and regulators	Most likely scenario for TACT is demonstration of non-inferior efficacy, slightly worse tolerability and evidence indicating absence of clinically important and frequent safety signals compared to standard ACT. At the same time the prevention of selection of drug resistance is unlikely to be demonstrated by individual clinical trials programs.	These key features are an uncomfortable combination for funders, pharmaceutical companies and regulators alike as no clear individual benefit for patients treated with TACT may be demonstrated in individual clinical development plans. A balanced decision needs to be taken to determine whether triple or multidrug combinations should become the new treatment paradigm (in analogy to HIV and Tb where drug resistance poses a similarly important threat) or not on the assumption that TACT would more effectively reduce the risk for the selection of drug resistance than current ACT. This may come with a slightly less favourable tolerability profile than current ACT. At the same time the potential benefit of TACT on the potential to reduce the emergence of drug resistance is unlikely to be documented in the short run. TACT’s full potential may therefore only be appreciated when aggregating data over a longer period of time after implementation. This is a difficult decision for policy makers, regulators, funders but needs to be taken consciously accepting in all honesty this strategic dilemma.

Pharmaceutical companies have been pivotal in the development of new anti-malarials including the most widely used combination therapies artemether-lumefantrine, dihydroartemisinin–piperaquine, artesunate–amodiaquine or more recently pyronaridine-artesunate. However, it may be argued that in the past it was mostly academic institutions in collaboration with the World Health Organization driving new therapeutic concepts for treatment of malaria. This includes the shift from anti-malarial monotherapies to ACT, as well as the subsequent global ban of artesunate monotherapy [4, 5, 22]. A decision to recommend a switch from ACT to TACT will be a strategic choice based on the understanding of the history of anti-malarial therapy and evolving evidence from clinical trials. This decision will have to be balanced on the benefit of individual patients in terms of efficacy, tolerability, and safety of first-line anti-malarial drug regimens and the interest of future patients by maximizing the impact of first line treatments on the prevention of drug resistance. Even if direct benefit may not be seen in a single clinical trial or clinical development programmes, it should be a conscious decision to embark on this treatment paradigm. This holds true for TACT as well as for other novel anti-malarial drugs of new classes currently in the clinical development pipeline that may be used in anti-malarial combinations of two or more drugs.

In summary, clinical researchers, funders, regulators and the public health community have to wholeheartedly accept a difficult situation and need to be open to unconventional choices. Current clinical development programmes for TACT will most likely not be able to prove superior efficacy or equal tolerability compared to currently used ACT. Similarly, it will prove difficult to demonstrate their potential to prevent the development of drug resistance in individual clinical trials. Based on the lessons learned over the past century and the analogies to antimicrobial drug resistance, TB and HIV, it may still be the right time and the right place to continue the development of TACT while accepting that convincing evidence for or against their use will have to be assessed over the long run.

## Data Availability

Not applicable.
